# Semi-supervised Telerehabilitation Exercise Program for Pelvic Floor Function in Pregnant Women with Gestational Diabetes Mellitus: A Randomized Controlled Clinical Trial

**DOI:** 10.63144/ijt.2026.6738

**Published:** 2026-06-01

**Authors:** Joyce Maria Pereira de Oliveira, Letícia Amaro Vieira, Edson Silva-Filho, Adriana Gomes Magalhães, Leony Morgana Galliano, Eduardo Caldas Costa, Sávio Ferreira Camargo, Maria Thereza Albuquerque Barbosa Cabral Micussi

**Affiliations:** 1 Postgraduate Program in Applied Sciences to Women’s Health, Federal University of Rio Grande do Norte, Natal, Brazil; 2 Department of Physiotherapy, Federal University of Rio Grande do Norte, Natal, Brazil; 3 Department of Physical Education, Federal University of Rio Grande do Norte, Natal, Brazil; 4 Postgraduate Program in Applied Health Sciences, Federal University of Rio Grande do Norte, Natal, Brazil

**Keywords:** Gestational diabetes mellitus, Exercise, Pelvic floor, Telerehabilitation, Urinary incontinence

## Abstract

**Introduction:**

Pelvic floor dysfunctions (PFD) are common during pregnancy, and the metabolic stress of Gestational Diabetes Mellitus (GDM) may exacerbate this condition. Supervised muscle training is an option that might be effective for treating PFD. This study aimed to evaluate the effects of a semi-supervised exercise program, delivered via telerehabilitation, on pelvic floor function in pregnant women with GDM.

**Methods:**

A randomized, controlled, blinded clinical trial was conducted with participants allocated into an Exercise Group (EG; n=20) and a Control Group (CG; n=20). The EG performed an exercise protocol (aerobic, global strength, and pelvic floor muscle–specific training) for 10–20 weeks, with telemonitoring via smartphone. The CG received educational booklets. Pelvic floor muscle function, PFD symptoms and impacts, and sexual function were assessed before and after the intervention. Perceived global improvement was measured at the end of the study.

**Results:**

Although no between-group differences were observed post-intervention for the overall sample, the stratified analysis of participants with urinary incontinence (UI) revealed a significant time-by-group interaction for the International Consultation on Incontinence Questionnaire–Short Form (ICIQ-SF) (p = 0.007). Among women with UI, post-intervention between groups comparisons indicated mean differences of 4.07 (IC95%: 1.03;7.11) for the ICIQ-SF, 14.51 (IC95%: 1.26;27.76) for the UDI, and 2.68 (IC95%: −19.21;24.57) for the UIQ. In this subgroup, the EG demonstrated a numeric significant reduction in urinary discomfort and a protective effect against worsening UI severity compared with the CG.

**Conclusion:**

A semi-supervised telerehabilitation program appears to be effective in reducing and preventing the progression of urinary symptoms in pregnant women with GDM and pre-existing urinary incontinence.

Functional alterations in the pelvic floor muscles (PFM) are common during pregnancy and are associated with physiological changes such as increased intra-abdominal pressure and hormonal fluctuations, which may compromise their supportive and sphincteric control capacity (Dietz, 2019; Haylen et al., 2010). These muscles play a fundamental role in maintaining urinary and fecal continence, supporting the pelvic organs, and contributing to sexual function (Dietz, 2019; Haylen et al., 2010).

Pregnant women with metabolic comorbidities, particularly those with gestational diabetes mellitus (GDM), exhibit a greater predisposition to pelvic floor dysfunction (PFD) (Kocaöz et al., 2020; Wang et al., 2023). The global prevalence of GDM is approximately 14%. It is important to note that, in Brazil, meta-analytic data reported a similar pooled prevalence (Dall et al., 2021; Vasconcelos et al., 2024). Gestational hyperglycemia may induce microvascular and neuropathic changes, compromising muscle fiber integrity and local innervation and thereby increasing the risk of PFD (Kocaöz et al., 2020; Wang et al., 2023). Consequently, pregnant women with GDM are exposed to both mechanical overload and metabolic stress on the musculature, reinforcing the need for effective and applicable interventions.

Pelvic floor muscle training (PFMT) is the primary conservative strategy recommended to improve muscle function, supported by level 1A evidence (Dumoulin et al., 2018; Kegel, 1948). The effectiveness of PFMT depends on adherence and correct exercise performance. Studies have shown that supervised programs, whether in person or remote, yield better outcomes than unsupervised practice (Fitz et al., 2017).

Given the challenges associated with adherence to fully in-person programs, telerehabilitation has emerged as an effective strategy. A meta-analysis demonstrated that, for musculoskeletal conditions, real-time remote rehabilitation is as effective as in-person interventions, with comparable clinical outcomes and adherence rates (Carter et al., 2018). This evidence suggests that telerehabilitation may be a viable alternative for high-risk pregnant women who require continuous monitoring but face logistical barriers. Semi-supervised models that combine in-person sessions with remote follow-up have the potential to optimize training effects and improve adherence (Carter et al., 2018). However, the effectiveness of this approach in pregnant women with GDM and urinary incontinence (UI) symptoms remains insufficiently explored. We hypothesized that a semi-supervised telerehabilitation exercise program would improve pelvic floor function, reduce urinary symptoms, and enhance sexual function, while also demonstrating high adherence among participants.

Considering these assumptions, this study aimed to evaluate the effects of a semi-supervised telerehabilitation exercise program on pelvic floor function in pregnant women with GDM.

## Methods

### Study Design and Ethical Considerations

This was a single-blind, randomized, controlled clinical trial that followed CONSORT guidelines (Hopewell et al., 2025). Pregnant women diagnosed with GDM and attending the high-risk prenatal care outpatient clinic of Maternidade Escola Januário Cicco (MEJC/EBSERH), in Natal, Brazil, were recruited between September 2024 and May 2025. The study complied with the Declaration of Helsinki and was approved by the Research Ethics Committee of the Federal University of Rio Grande do Norte (Protocol No. 6.613.712). All participants provided written informed consent. Prior to study initiation, the trial was registered in the Brazilian Registry of Clinical Trials (ReBEC: RBR-9qm8wqh).

### Eligibility Criteria

The following inclusion criteria were adopted: (1) age between 18 and 45 years; (2) diagnosis of GDM according to World Health Organization (2013), with fasting blood glucose ≥ 92 and ≤ 125 mg/dL; (3) gestational age between 14 and 25 weeks; (4) medical clearance to perform physical exercise; (5) PFM strength ≥ 2 on the Modified Oxford Scale (MOS); and (6) access to a smartphone. Exclusion criteria were: (1) prescription of insulin therapy; (2) multiple pregnancy; (3) comorbidities such as chronic hypertension or preeclampsia; (4) history of fetal death; (5) recurrent miscarriages (>2); (6) orthopedic limitations; and (7) alcohol consumption (≥2 alcoholic drinks per week) or tobacco use (World Health Organization, 2013; Zajdenverg et al., 2023).

### Interventions

**Figure 1:**
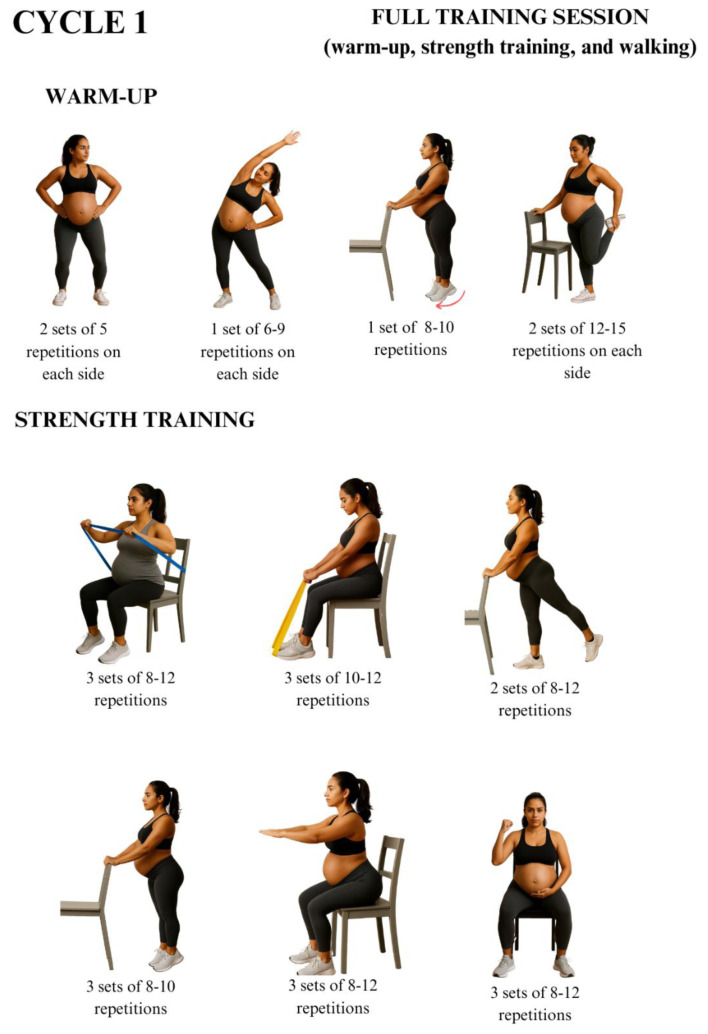
Cycle 1 of the Semi-supervised Telerehabilitation Exercise Program

**Figure 2:**
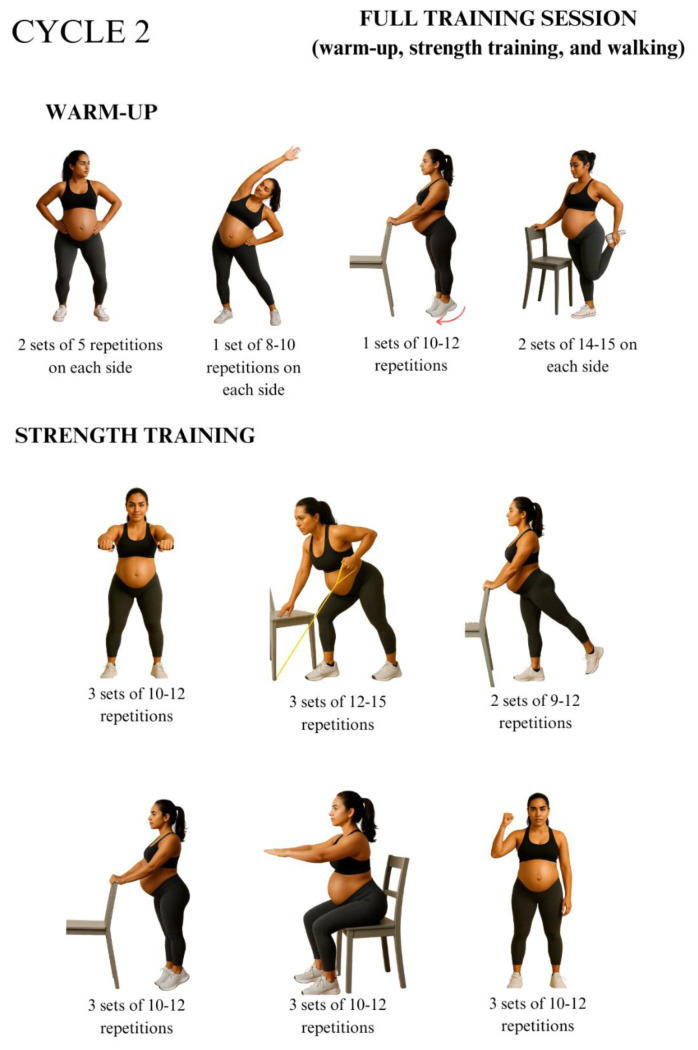
Cycle 2 of the Semi-supervised Telerehabilitation Exercise Program

The Exercise Group (EG) participated in a semi-supervised training program lasting 10 to 20 weeks. Sessions were held three times per week, lasting 40 to 55 minutes, and consisted of aerobic exercise (walking), global strengthening exercises (upper and lower limbs), and specific PFMT (rapid contractions). The protocol was designed according to the Canadian Guideline for Physical Activity Throughout Pregnancy (Mottola et al., 2018), the American College of Obstetricians and Gynecologists (2020), and the Brazilian Physical Activity Guidelines (Mielke et al., 2021). Progression between cycles was achieved by increasing the minimum number of repetitions, external load or resistance, and by changing exercise positioning from seated to standing, as detailed in Figures 1 and 2.

Exercise monitoring and reminders were delivered via smartphone using WhatsApp®, through text, audio, and video messages sent by the research team, identified as “Rosa.” Participants received one reminder before and one after each session on pre-scheduled training days. Adverse events, session completion rate, and adherence to the training program were tracked.

All EG participants attended an in-person session to receive exercise videos, become familiar with the activities, and clarify doubts. Additional in-person meetings occurred between 29+0 and 30+6 weeks of gestation to deliver new exercise videos following the planned progression. All supervised sessions were conducted by two trained researchers and an experienced physical educator from the institution (over 10 years of experience).

The CG received three educational booklets developed by the research team, containing information about GDM, pelvic floor health, and physical activity, based on the Brazilian Physical Activity Guidelines (Mielke et al., 2021). Both groups continued receiving routine care from the high-risk prenatal service.

### Assessment and Outcomes

The initial assessment, at gestational age between 14 and 25 weeks, included the collection of sociodemographic, clinical, gynecological, and obstetric data, such as age, educational level, marital status, pre-pregnancy body mass index (BMI), gestational age, history of fetal macrosomia, history of GDM in previous pregnancies, vaginal delivery, and number of pregnancies. The following questionnaires were also administered: Pelvic Floor Distress Inventory-20 (PFDI), Pelvic Floor Impact Questionnaire-7 (PFIQ), International Consultation on Incontinence Questionnaire - Short Form (ICIQ-SF), and Female Sexual Function Index (FSFI). Pelvic floor (PF) strength and pressure were assessed by vaginal manometry with the Peritron™ model 9300AV, and by vaginal palpation using the MOS respectively. The MOS classifies muscle strength into six grades, from 0 (no muscle response) to 5 (strong contraction with firm compression of the examiner’s fingers and positive movement toward the pubic symphysis) (Ferreira et al., 2011) and was used solely as an eligibility criterion. Vaginal manometry was performed with the participant in the supine position, knees flexed, and feet supported on the gynecological examination table. Participants were instructed to perform three maximum voluntary PFM contractions.

The final assessment took place between 35 and 36 weeks’ gestation. Participants underwent a physical examination and completed the same questionnaires as at baseline, in addition to the Patient Global Impression of Change (PGI-C). All assessments were conducted by an experienced, highly trained physiotherapist who was independent from the intervention team, and all procedures took place at MEJC/EBSERH.

Initially, the primary outcome of the study was pelvic floor function, measured by PFM pressure, urinary symptoms, and sexual function. PFM pressure was assessed by vaginal manometry, and the value used in the analysis was the mean of the three recorded contractions, expressed in cmH_2_O (Ferreira et al., 2011). In the final assessment (35–36 weeks’ gestation), a low adherence rate was observed, with 19 participants refusing the manometry assessment. Thus, urinary symptoms were considered the primary outcome.

Symptoms and the impact of PFD were assessed using the PFDI and PFIQ. The PFDI measures the presence and discomfort of urinary, colorectal, and pelvic organ prolapse symptoms, while the PFIQ evaluates the functional impact of these symptoms on daily activities, social relationships, and emotional aspects (Arouca, 2015). Analyses included total scores and the specific urinary subscales of both questionnaires: the Urinary Distress Inventory (UDI) and the Urinary Impact Questionnaire (UIQ) (Arouca, 2015). The ICIQ-SF was also used to quantify UI severity and its impact on quality of life. For all questionnaires and subscales, higher scores indicate greater severity or negative impact (Tamanini et al., 2004).

Other outcomes assessed sexual function and global impression of change. Sexual function was assessed using the FSFI, which evaluates desire, arousal, lubrication, orgasm, satisfaction, and pain. Higher scores indicate better sexual function, and scores ≤ 26.55 suggest dysfunction (Rosen et al., 2000). The PGI-C captured patients’ subjective perception of improvement or worsening since the beginning of the study, with higher scores indicating greater perceived improvement (Yalcin & Bump, 2003).

### Sample Size Calculation

The sample size was calculated a priori using G*Power (version 3.1.9.2), with vaginal manometry defined as the original primary outcome. The estimation was based on an F test for repeated-measures ANOVA (within–between interaction), assuming a significance level of 0.05, 80% statistical power, two groups, two measurement points, nonsphericity correction 1, and an effect size of 0.23 (Oliveira Bezerra et al., 2024). This procedure indicated that a minimum of 40 participants was required; therefore, 40 individuals were recruited and randomized into two groups of 20.

### Randomization, allocation concealment and Blinding

A total of 40 participants were recruited and randomly allocated into two groups: Exercise Group (EG) and Control Group (CG), with 20 women each. Within each group, participants were stratified according to the presence of UI, identified using the ICIQ-SF.

Randomization was performed electronically by an independent researcher not involved in assessments or interventions, using the website randomization.com, which assigned each participant to the EG or CG with equal probability. Allocation sequences were generated in blocks of four (2:2 ratio), stratified by age group (18–29 years and 30–45 years). To ensure blinding, the randomization process and participant training were conducted by researchers 1 and 2, whereas baseline and post-intervention assessments were performed by researcher 3. Data analysis was carried out by researcher 4. Allocation concealment was ensured using opaque, sealed, sequentially numbered brown envelopes, which were opened only after confirming participant eligibility and obtaining informed consent.

### Statistical Analysis

Data were analyzed using Jamovi software (version 2.7.5.0). Sociodemographic variables: including age, pre-pregnancy BMI, gestational age, education, race or ethnicity, marital status, history of fetal macrosomia, previous GDM, type of delivery, and number of pregnancies, were described using means and standard deviations for continuous data, and percentages for categorical variables. Baseline comparisons between groups were performed using Student’s t-tests for continuous variables and chi-square tests for categorical variables.

To analyze the outcomes, we used Generalized Estimating Equations (GEE). The models included group (exercise group vs. control group), time (assessment points), and the group × time interaction as independent variables. The interaction term was used to evaluate whether changes over time differed between groups. We inserted a random effect for the dataset intercept and fixed effects for group, time, and group × time interaction. The dependent variables were vaginal manometry, Pelvic Floor Impact Questionnaire (PFIQ), Pelvic Floor Distress Inventory (PFDI), International Consultation on Incontinence Questionnaire–Short Form (ICIQ-SF), and Female Sexual Function Index (FSFI) scores. Participants were further stratified according to the presence or absence of UI identified by the ICIQ-SF, to evaluate post-intervention differences between groups regarding urinary symptoms measured by the ICIQ-SF, UDI, and UIQ.

Model adequacy was assessed through Q–Q plots, histograms, and residual analysis. Additional goodness-of-fit indices such as A/C, B/C, Q/C, chi-square/df ratio, and intraclass correlation coefficient were used to confirm the inclusion of fixed factors. GEE analyses were conducted at baseline and post-intervention. PGI-C responses were categorized into three levels (positive opinion, no change, negative opinion) and analyzed using chi-square tests.

Results were expressed as means, confidence intervals, and p-values, with statistical significance set at p < 0.05. Intention-to-treat analysis was applied when appropriate, with missing data replaced by the last observation carried forward. For the PGI-C, missing responses were classified as “no change.”

## Results

**Figure 3:**
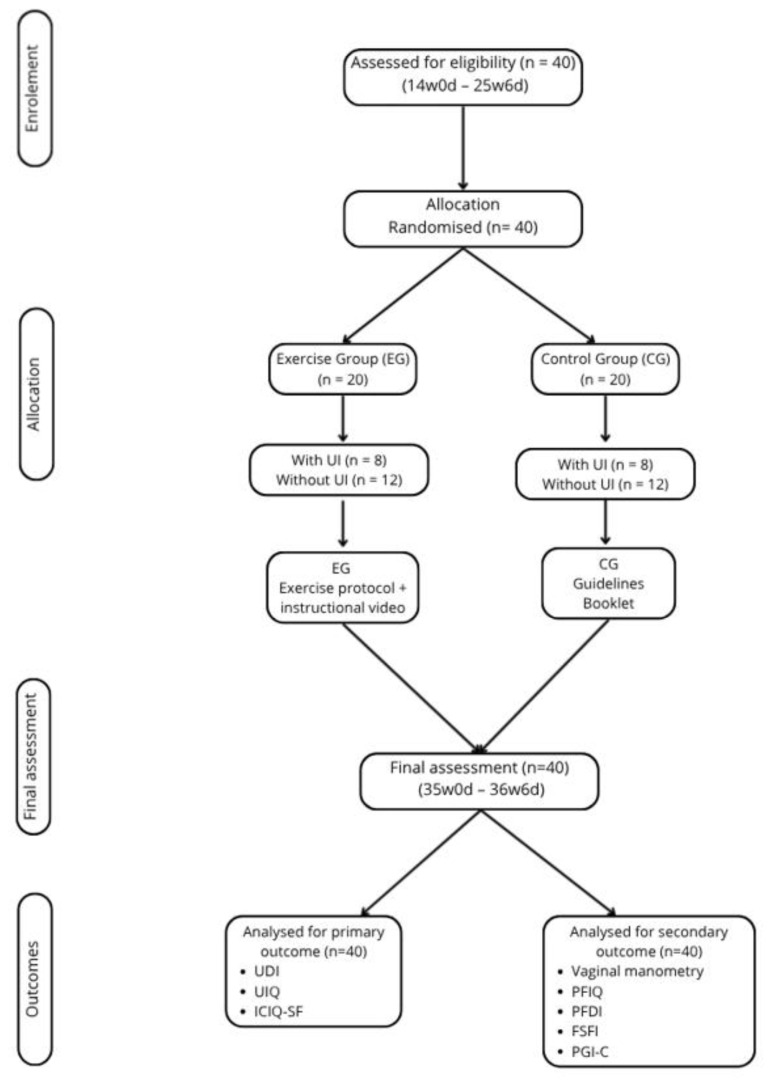
Flow Diagram *Note*: EG = Exercise Group; CG = Control Group; UI = Urinary Incontinence; PFDI = Pelvic Floor Distress Inventory-20; PFIQ = Pelvic Floor Impact Questionnaire-7; ICIQ-SF FSFI = Female Sexual Function Index; UDI = Urinary Distress Inventory; UIQ = Urinary Impact Questionnaire; PGI-C = Patient Global Impression of Change.

A total of 40 participants were analyzed, as shown in Figure 3. Table 1 presents the sociodemographic and clinical characteristics of the sample. The mean gestational age at inclusion was similar between groups (22.3 ± 2.9 weeks in the EG and 24.1 ± 3.3 weeks in the CG). Most participants lived with a partner, had completed secondary education, and self-identified as mixed-race. A high prevalence of previous GDM and fetal macrosomia was also noted in both groups, in addition to a predominance of multiparous women (80% in the EG and 65% in the CG). There were no differences between the groups at baseline assessment, except for age (p = 0.02). As the groups were randomized, this imbalance is likely attributable to chance rather than a systematic issue.

Overall, two participants from each group discontinued participation. In the CG, two participants experienced adverse outcomes, including stillbirth associated with preterm birth. Furthermore, some vaginal manometry data were not collected, nine from the EG and eight from the CG, due to participant refusal to undergo the assessment in the third trimester.

In the between-group comparisons, no statistically significant differences were observed in either group for vaginal manometry, PFIQ, PFDI, ICIQ-SF, and FSFI scores (p > 0.05 for all comparisons), as detailed in Table 2.

When participants were stratified according to the presence or absence of UI, a significant interaction was identified among women with UI over time for ICIQ-SF (p = 0.007). Among women with UI, post-intervention comparisons between the EG and CG showed mean differences of 4.07 for the ICIQ-SF, 14.51 for the UDI, and 2.68 for the UIQ. Table 3 presents the corresponding means and confidence intervals at baseline, post-intervention, and the within-group changes.

At the end of interventions, a statistically significant difference was observed between groups in PGI-C responses (χ^2^ = 7.23; p = 0.02). In the EG, most participants, (85%, n=17), reported improvement, while (15%, n = 3) reported no change and none reported worsening. In contrast, less than half of the CG participants (45%, n = 9) reported improvement, half (50%, n = 10) reported no change, and 1 (5%) indicated worsening.

## Discussion

This study aimed to evaluate the effects of a semi-supervised telerehabilitation exercise program on pelvic floor function in pregnant women with GDM, considering urinary symptoms, PFM pressure, and sexual function. Results from the stratified analysis of participants with and without UI indicated a positive impact on urinary symptoms, such as reduced urinary frequency, improved urgency, and fewer episodes of involuntary urine loss after the intervention among women with UI.

At the end of the intervention, the EG demonstrated significantly lower UDI scores compared to the CG, reinforcing the benefit of the intervention. In contrast, the CG not only failed to improve but also showed a tendency toward worsening symptoms. These findings highlight the positive and protective effects of the telerehabilitation exercise protocol in the group with UI, suggesting improved muscle function and preservation of pelvic floor pressure. This maintenance is particularly relevant considering that the literature indicates that pregnant women tend to experience overload of this musculature in late pregnancy, which may favor the development of PFD, including urinary complaints (Cattani et al., 2024; Van Veelen et al., 2014).

Despite the absence of statistically significant differences in UI severity and its impact on quality of life between groups, a mean reduction of 2.03 points in the ICIQ-SF score was observed for women with UI in the EG, close to the 2.5-point threshold considered clinically relevant in the literature (Haley et al., 2008; Samsa et al., 1999). Conversely, participants who received only the educational booklet showed a statistically significant increase in symptom severity (p = 0.03), indicating clinical worsening. These findings suggest that CG participants experienced greater impairment in quality of life at the end of pregnancy. Thus, the semi-supervised exercise protocol with telemonitoring may have not only reduced urinary complaints but also positively impacted the quality of life in the EG. These results align with evidence from a systematic review with meta-analyses showing that digital care programs, through apps, web platforms, and other digital tools, promote significant reductions in UI symptoms measured by the ICIQ-SF, as well as improve quality of life and treatment adherence (Hao et al., 2024).

Regarding the functional impact of UI on daily activities, social relationships, and emotional aspects, clinical improvement was observed. The EG showed an approximate 70% reduction in UIQ scores, whereas the CG demonstrated a smaller improvement (57.7%). Interestingly, the intervention group had higher baseline scores, indicating greater initial impairment. This finding may suggest that the greater functional impact perceived by EG participants facilitated increased engagement with the protocol, resulting in more expressive benefits after the intervention. This interpretation is consistent with evidence indicating that greater adherence improves PFMT efficacy (Wang et al., 2024) and that symptom burden modulates both engagement and treatment response in digital pelvic floor interventions (Hao et al., 2024).

Our findings indicate that supervised exercise combined with telemonitoring is a promising therapeutic strategy for managing PFD in this population. A longitudinal study conducted by Weinstein et al. (2025), although in non-pregnant women, demonstrated the long-term sustainability of telerehabilitation effects, showing that a home biofeedback device improved UI symptoms for up to 24 months. The literature suggests that telerehabilitation is effective for managing PFD, enhances treatment adherence, and optimizes outcomes among individuals with limited access to in-person therapy (Weinstein et al., 2025).

Regarding satisfaction levels, most women in the EG reported global improvement after the intervention. These findings align with evidence indicating that PFMT interventions delivered through digital technologies or hybrid models show high satisfaction rates, good adherence, and clinical effectiveness comparable to in-person programs. A recent meta-analysis demonstrated that PFMT programs delivered via telerehabilitation are feasible, effective, and well tolerated, with high acceptance among participants (Wang et al., 2024). Additionally, the use of the PGI-C, a validated and widely adopted instrument to evaluate patient-reported global impression of change, reinforces the importance of subjective perception as a meaningful clinical outcome (Yalcin & Bump, 2003). In this context, the semi-supervised model offered greater flexibility and accessibility for high-risk pregnant women, maintaining clinical safety, promoting adherence, and enhancing therapeutic outcomes.

Although the literature recommends pelvic floor assessment using intravaginal methods such as vaginal manometry (Apostolakis-Kyrus et al., 2021; Barbosa et al., 2024; Pires et al., 2020), a substantial proportion of participants did not complete this examination at the end of pregnancy, requiring the use of an intention-to-treat approach for several participants. To address this limitation, the study relied on validated urinary symptom questionnaires, which were already part of the protocol and provide an indirect yet clinically meaningful indicator of pelvic floor function, as lower urinary symptom burden is associated with better pelvic floor performance. The approach to handling missing data may have influenced the results and should be considered when interpreting the findings.

## Conclusion

The semi-supervised exercise protocol delivered via telemonitoring presented a trend toward improving urinary symptoms and exerting a protective effect against symptom progression in pregnant women with GDM and pre-existing UI. The protocol proved to be a viable strategy adjunct to usual care for this high-risk population, reinforcing the importance of active, semi-supervised interventions in promoting pelvic health during pregnancy. It is important to note that no statistical differences between groups were detected, therefore, these results should be interpreted with caution.

## Figures and Tables

**Table 1:** Intergroup Sociodemographic Characteristics of Women with Gestational Diabetes

Sociodemographic variables	Exercise Group (n = 20)	Control Group (n = 20)
Age (years)^a^	32.5 ± 5.40	28.4±6.23
Pre-gestational BMI (kg/m^2^)^a^	20.6±4.56	29.8±7.56
Gestational age^a^	22.3±2.90	24.1±3.30
Schooling^b^		
High school or lower	15 (75%)	16 (80%)
Some college or higher	5 (25%)	4 (20%)
Race/Ethnicity^b^		
White	2 (10%)	6 (30%)
Asian	1 (5%)	0
Black	4 (20%)	3 (15%)
Indigenous	1 (5%)	0
Brown	12 (60%)	11 (55%)
Marital status^b^		
Single/Not married	17 (85%)	18 (90%)
Partnered	3 (15%)	2 (10%)
History of fetal macrossomia^b^		
Yes	18 (90%)	18 (90%)
Previous gestational diabetes		
Yes	19 (95%)	19 (95%)
Vaginal delivery^b^		
0	11 (55%)	13 (65%)
1	8 (40%)	2 (10%)
2	1 (5%)	5 (25%)
Number of pregnancies^b^		
1	4 (20%)	7 (35%)
≥ 2	16 (80%)	13 (65%)

*Note*.

a continuous data represented as mean and standard deviation;

b categorical data represented as total quantity and percentage.

**Table 2:** Between and Within Group Analysis of the Manometry, PFIQ, PFDI, ICIQ-SF, and FSFI

Functional test	Exercise Group (n = 20)	Control Group (n = 20)	p-value between-groups
**Manometry** a			0.63
Baseline	25.70 (19.80;31.60)	27.40 (21.50;33.20)	
Final	26.80 (20.90;32.60)	28.90 (23.10;34.80)	
Within-group change	26.20 (20.60;31.90)	28.10 (22.50;33.80)	
**PFIQ** a			0.97
Baseline	40.20 (19.98;60.40)	28.60 (8.33;48.80)	
Final	19.60 (−0.66;39.80)	30.50 (10.23;50.70)	
Within-group change	29.90 (11.60;48.20)	29.50 (11.20;47.80)	
**PFDI** a			0.88
Baseline	54.0 (36.90;71.0)	46.60 (29.40;63.40)	
Final	40.90 (23.80;57.90)	45.10 (28.10;62.10)	
Within-group change	47.40 (31.70;63.10)	45.80 (30.10;61.40)	
**ICIQ-SF** a			0.77
Baseline	4.70 (1.97;7.43)	3.50 (0.77;6.23)	
Final	3.90 (1.17;6.63)	4.15 (1.42;6.88)	
Within-group change	4.30 (2.0;6.60)	3.82 (1.53;6.12)	
**FSFI** a			0.56
Baseline	22.60 (18.90;26.20)	25.20 (21.60;28.90)	
Final	21.60 (17.90;25.20)	21.40 (17.80;25.10)	
Within-group change	22.10 (19.0;25.10)	23.30 (20.30;26.40)	

*Note*.

a data expressed as mean and 95% confidence interval.

PFIQ: Pelvic Floor Impact Questionnaire short PFDI: Pelvic Floor Distress Inventory. ICIQ-SF: International Consultation on Incontinence Questionnaire; FSFI: Female Sexual Function Index.

**Table 3:** Comparison of Urinary Outcomes between the Active and Control Groups, Stratified by the Presence of Baseline Urinary Incontinence

Functional test	Exercise Group + UI (n = 8)	Exercise Group + No UI (n =12)	Control Group + UI (n = 8)	Control Group + No UI (n = 12)
**ICIQ-SF**				
Baseline	11.73 (9.78.13.69)	0 (−1.59;1.60)	8.74 (6.79;10.70)	0 (−1.59;1.60)
Final	9.71 (7.76;11.67)	0 (−1.57;1.62)	13.78 (11.53;16.05)	0 (−1.46;1.50)
Within-group change	10.72 (9.31;12.15)	0 (−1.15;1.18)	11.26 (9.74;12.79)	0 (−1.11;1.14)
**UDI**				
Baseline	37.80 (29.22.46.4)	9.98 (2.89;17.1)	35.06 (26.48;43.60)	12.60 (5.51;19.70)
Final	24.36 (15.77;32.90)	8.80 (1.71;15.90)	38.87 (29.02;48.70)	10.49 (3.89;17.10)
Within-group change	31.08 (24.33;37.80)	9.39 (3.70;15.10)	36.96 (29.77;44.20)	11.54 (6.03;17.10)
**UIQ**				
Baseline	27.90 (13.50;42.30)	5.96 (−6.47;18.40)	10.80 (−3.55;25.20)	13.42 (0.98;25.80)
Final	7.25 (−7.12;20.60)	9.04 (−3.40;21.50)	4.57 (−11.50;20.60)	16.05 (4.21;27.90)
Within-group change	17.59 (5.12;30.10)	7.50 (−3.61;18.60)	7.68 (−5.35;20.70)	14.73 (3.85.25.60)

*Note.* Values presented as mean and confidence interval. UI: Urinary Incontinence. ICIQ-SF: International Consultation on Incontinence Questionnaire-Short Form; UDI: Urinary Distress Inventory; UIQ: Urinary Impact Questionnaire.
